# Stress and Nursing Home Adjustment: Mediation by Resourcefulness and Self-Efficacy

**DOI:** 10.1093/geroni/igaa057.596

**Published:** 2020-12-16

**Authors:** Huimin Xiao, Binbin Yong

**Affiliations:** Fujian Medical University, Fuzhou, China

## Abstract

Relocation to a nursing home is often assumed to be associated with stress for older adults. This study aimed to explore how stress affect psychological adjustment of nursing home residents. A cross-sectional survey was conducted. A sample of 386 nursing home residents was recruited from 11 nursing homes in Fujian Province, Southeast China. The Nursing Home Adjustment Scale, Perceived Stress Scale, Resourceful Scale, and General Self-Efficacy Scale were adopted to collect data. The path analysis was used to analyze the relationship of stress, nursing home adjustment, resourcefulness, and self-efficacy. The results indicated that stress directly caused poor nursing home adjustment. It also indirectly affected nursing home adjustment through the mediators of resourcefulness and self-efficacy, respectively. Furthermore, chained mediation was found from stress to nursing home adjustment through resourcefulness, and then through self-efficacy to cope with the negative affect. The current study contributes to the understanding of the mechanism of stress on nursing home adjustment in older adults. To improve their psychological adjustment, additional focus should be placed on enhancing resourcefulness and self-efficacy in nursing home residents.

